# Energy Expenditure of Special Forces Soldiers in Relation to Equipment Load and Movement Speed

**DOI:** 10.3390/nu18010027

**Published:** 2025-12-20

**Authors:** Emilian Zadarko, Patryk Marszałek, Maria Zadarko-Domaradzka, Beata Penar-Zadarko, Krzysztof Przednowek

**Affiliations:** 1 Faculty of Physical Culture Sciences, Collegium Medicum, University of Rzeszow, 35-959 Rzeszow, Poland; pmarszalek@ur.edu.pl (P.M.); mzadarko@ur.edu.pl (M.Z.-D.); krprzednowek@ur.edu.pl (K.P.); 2 Faculty of Health Sciences and Psychology, Collegium Medicum, University of Rzeszow, 35-959 Rzeszow, Poland; bpenar@ur.edu.pl

**Keywords:** energy expenditure, VO_2_max, military special forces, soldiers, Cosmed K5

## Abstract

**Background/Objectives:** Additional load is associated with a significant increase in energy expenditure during soldiers’ movement. The level of energy expenditure during military tasks depends on the speed at which soldiers move. The aim of the study was to determine how the speed of movement and military load in the form of a 20-kg backpack affect the energy expenditure of special forces operators. **Methods:** The study included a group of 24 special forces operators. The energy expenditure of participants was measured using a portable Cosmed K5 gas analyzer operating in “breath-by-breath” mode. Energy expenditure was calculated based on VO_2_ and VCO_2_ data. Respiratory exchange ratio (RER) was recorded in parallel with VO_2_ and VCO_2_ and used to calculate the oxidation of energy substrates. The soldiers moved in 6-min intervals at the following speeds: 4.5 km/h, 6.5 km/h, 8.5 km/h, 10.5 km/h. First, the soldiers covered each speed without a load, and then with a 20 kg tactical military backpack. **Results:** The analysis showed that increasing speed and the use of an external load significantly increased all physiological and metabolic responses. Speed × load interactions were observed for some metabolic variables, whereas no such interactions were found for heart rate. **Conclusions:** Adding a 20 kg tactical backpack causes a significant increase in energy expenditure at all speed levels. The additional load resulted in an average increase of 10% in heart rate (%HRmax) and 20% in oxygen uptake (%VO_2_max). A more than threefold increase in energy expenditure was recorded (14.77 kcal/min without load and 17.70 kcal/min with a backpack at a speed of 10.5 km/h vs. 5.01 kcal/min and 6.35 kcal/min at a speed of 4.5 km/h).

## 1. Introduction

Military service requires soldiers to have not only tactical and psychological skills, but above all a high level of physical fitness [1,2]. Active duty soldiers must be prepared to perform tasks in difficult terrain conditions with limited sleep and access to food and water [3,4]. For this reason, physical fitness is one of the basic components of military training [5,6]. Issues related to energy expenditure, the energy value of food, and, consequently, the energy balance of soldiers are also of particular importance [7].

A key factor that can significantly reduce the effectiveness of tasks is additional equipment, which is a basic element of every soldier’s gear [8,9,10]. It includes, among other things, weapons, ammunition, a tactical vest, a helmet, and a military rucksack [11]. The weight of this equipment can be as high as 60% of a soldier’s body weight [12]. Previous studies indicate that the weight of soldiers’ equipment may vary depending on the specific military formation and the type of tasks performed [13]. The average weight of assault soldiers’ equipment is 15–18 kg [14], while American soldiers in Afghanistan carried an average of about 22 kg, and in emergency situations, this weight could reach up to 34 kg [15].

Apart from the weight of tactical equipment, its distribution is also an important factor. Carrying a load close to the center of gravity minimizes energy expenditure and allows for a more upright posture, similar to that observed without an additional load [16]. Some studies indicate that carrying weight both in front and behind the body promotes its even distribution, which translates into better dynamic stability and movement control [17].

In the case of carrying a heavy backpack, the distribution of equipment inside it is crucial. Placing the heaviest items at the top of the backpack can reduce stability and increase the load on the musculoskeletal system, especially when moving on terrain with varying slopes [18]. Carrying a backpack in which the heaviest weight is close to the body’s center of gravity, using hip belts and shorter, stiffer shoulder straps, provides more favorable conditions for maintaining balance, optimal muscle activation, and energy efficiency [16,17,18,19,20].

The additional load is associated with a significant increase in energy expenditure during soldiers movement [21,22]. Military equipment leads to a slowdown in movement and increased metabolic costs [9,23]. These costs may vary and depend on the nature of the tasks performed. The reference dietary allowance for US Army soldiers is approximately 3248 kcal/day, but studies have shown that during elite special forces training, the energy requirements can be significantly higher, reaching an average of 4204 kcal/day [24]. Other analyses have reported that during intensive combat courses, such as United States Marine Corps infantry officer training, energy expenditure reached as high as 5400 kcal/day [25].

The level of energy expenditure during military tasks depends on the speed at which soldiers move, which can be important in difficult terrain [16,26]. Previous studies have shown that during slow marching at a speed of 4 km/h, additional weight significantly increases metabolic cost [27]. Moreover, a load of 10–22 kg is sufficient to significantly increase energy expenditure and reduce the efficiency of soldiers’ movement [22,28]. Increased energy expenditure is associated with a physiological response of the cardiorespiratory system, which manifests itself in the form of increased oxygen consumption, heart rate, minute ventilation, and respiratory rate [22,26,29,30]. It is worth noting that when marching with a load exceeding 50% of body weight, the increase in oxygen consumption can rise from 25% to as much as 50% [31].

Current research findings indicate that with heavier loads, the metabolic cost of walking increases disproportionately, and the risk of musculoskeletal overload increases significantly [16]. What is more, the locomotion of soldiers includes not only walking but also running, which is crucial in emergency situations [11,32]. The severity of work, measured by energy expenditure, is an essential element in assessing physical load, which is the relationship between the demands of work in terms of physical effort and the capabilities of the body [7]. Previous studies have mostly focused on analyzing energy expenditure during marching, which does not fully reflect the actual conditions of soldiers’ movements [21,24,31,33].

However, it should be noted that Special Forces operators differ from conventional soldiers and are among the best-trained soldiers in the world, who voluntarily enlist in elite military units. The operational versatility of the tasks performed requires Operators to have a high level of motor skills, which are key to performing specific combat tasks [34].

Despite the available studies on external load and the way soldiers move, there is still a lack of clear scientific data precisely defining how additional weight affects the energy expenditure of soldiers.

The aim of the study was to determine how the speed of movement and military load in the form of a 20-kg backpack affect the energy expenditure of special forces operators. We assume that the increase in speed and the additional load of 20 kg will cause a significant, non-linear increase in energy expenditure and physiological load on soldiers, while shifting their metabolism towards greater use of carbohydrates.

## 2. Materials and Methods

### 2.1. Subject

The study included a group of 24 special forces operators who had completed a long-term training process and gave their written consent to participate in the study. Special forces soldiers were selected as an elite group for whom moving with an external load is an integral part of military service. The average age of the soldiers was 34.8 ± 4.0 years. Their average body height (BH) was 180.4 ± 5.0 cm and their average body weight (BW) was 85.5 ± 8.1 kg. The inclusion criteria were military service in an elite group of special forces operators, consent to participate, and no medical contraindications to participation in the study.

### 2.2. Procedure

The study was conducted in an athletics hall in stages over three consecutive weekdays. On the first day, medical examinations were conducted to clear the soldiers for exercise testing, and their consent was obtained. The study involved healthy soldiers in active military service, with no recent injuries, who were fully combat-ready. First, BH, BW, and body mass components were measured. Maximum oxygen uptake (VO_2_max) and heart rate (HRmax) were measured at the end of a progressive shuttle run test (20mSRT). The criteria for achieving VO_2_max were considered to be a VO_2_ plateau and respiratory exchange ratio (RER) > 1.10. The soldiers performed the test in sports clothing and footwear. These tests were also used to determine the percentage value of the internal load on the body (%HRmax and %VO_2_max) in subsequent stages of the tests. The following days (stages) of the tests consisted of the soldiers moving at different speeds. They moved in 6-min intervals at the following speeds: 4.5 km/h, corresponding to a leisurely walk, 6.5 km/h, corresponding to a brisk walk, 8.5 km/h, corresponding to a jog, and 10.5 km/h, corresponding to a run. The speeds were selected based on literature concerning the locomotion of soldiers carrying loads and the physiological costs of walking and running so that they correspond to typical forms of movement including walking, fast walking, jogging, and running [31,35,36,37]. First, the soldiers covered each speed without a load, and then with a 20 kg tactical military backpack. A 20 kg backpack is the standard load for special forces candidates during selection for these units in Poland. There was a one-day break between the test without a load and the test with a backpack load. The soldiers wore tactical military boots and field military uniforms. The military backpack was evenly packed and weighed. Each soldier individually adjusted the backpack to their personal settings.

The tests were part of a project carried out by the University of Rzeszów entitled “Identification and monitoring of health parameters in soldiers and uniformed services officers,” which was approved by the Bioethics Committee of the University of Rzeszów (approval code: 3/01/2021).

### 2.3. Anthropometric Measurements

Body height was measured without shoes in the so-called Frankfort Horizontal Plane position using a SECA 213 stadiometer (Hamburg, Germany) with an accuracy of 0.1 cm. Body weight was measured in light sportswear using a DC-360 analyzer (Tanita Corporation, Tokyo, Japan) in the morning. Body composition was estimated using Bioelectrical Impedance Analysis (BIA).

### 2.4. VO_2_ and HR Measurement

Direct measurement of maximum oxygen uptake VO_2_max, breath by breath, was performed using a k5 analyzer (Cosmed, Rome, Italy) during a 20-m shuttle run test (20mSRT) conducted in accordance with Leger’s description and procedures [38]. The initial speed of the 20mSRT was 8.5 km per hour. With each subsequent stage of the test, the speed increased by half a kilometer per hour. Before the test, the k5 analyzer was warmed up for a minimum of 60 min and then calibrated according to the recommendations using standard gases (16% O_2_ and 5% CO_2_). The analyzer was calibrated daily and before each subsequent test under the environmental conditions of the athletics hall (21.8 °C, 1004.25 hPa, and 57.4% humidity).

The size of the mask was adjusted individually before the first test and kept the same size during subsequent tests. Heart rate (HR) per minute during exercise was monitored using the Polar system (Polar Electro Oy, Kempele, Finland). The maximum HRmax value was considered to be the value reached at the end of the exercise. The exercise tests were performed two hours after a meal according to an individually planned time protocol.

### 2.5. Measurement of Energy Expenditure

The energy expenditure of participants was measured using a portable Cosmed K5 gas analyzer (Cosmed, Rome, Italy) operating in “breath-by-breath” mode. The system was fully calibrated before each test. The device recorded the volume of oxygen consumed (VO_2_) and carbon dioxide exhaled (VCO_2_); based on these values, energy expenditure (EE) was automatically calculated according to Weir’s formula [39]:

EE (kcal/min) = (3.941 × VO_2_ (L/min)) + (1.106 × VCO_2_ (L/min))

Each stage of exercise lasted 6 min, but only data from the last 5 min of each stage was included in the energy expenditure analysis. The first minute of each exercise was excluded as a transition period needed to reach a steady state of gas exchange. The analysis used average values from minutes 2–6, which allowed for stable and comparable energy expenditure results, which are expressed in kcal/min.

The respiratory exchange ratio (RER) was recorded in parallel with VO_2_ and VCO_2_ and used to calculate the oxidation of energy substrates. It was assumed that RER values between 0.70 and 1.00 indicate a dominant contribution of fats or carbohydrates as an energy source [40]. Data on CHO and Fat oxidation (g/min) were automatically generated by Omnia software version 2.3 (Cosmed) based on the average VO_2_ and VCO_2_ values from the last 5 min of the stage.

Energy expenditure was calculated from VO_2_ and VCO_2_ data, assuming a negligible contribution of protein oxidation according to a previous report [41].

### 2.6. Statistical Analysis

Before starting the study, a statistical power analysis was performed using G*Power 3.1.9.4 software. The analysis was performed for analysis of variance with repeated measures (ANOVA, within-subject design), taking into account 8 measurement conditions resulting from the study design (4 movement speeds × 2 load levels). A moderate effect size (f = 0.25), significance level α = 0.05, test power 1 − β = 0.80, correlation between repeated measures r = 0.5, and full sphericity (ε = 1) were assumed. Based on this, the minimum required sample size was 16 people.

Statistical analysis was performed using Jamovi software version 2.6.2. Descriptive statistics were presented as mean (M), standard deviation (Sd), minimum (Min) and maximum (Max) values. Next, the Shapiro–Wilk test was performed to verify the assumption of normal data distribution, which confirmed the normal distribution of the analyzed variables. In order to assess the relationship between the tested movement speeds of soldiers and external load, a two-way analysis of variance (ANOVA) was performed. The analysis covered both the differences between load conditions and between the movement speeds of the soldiers under study. A significance level of *p* < 0.05 was adopted. Effect sizes were assessed using partial eta squared (η^2^p), with values of 0.01, 0.06, and 0.14 indicating small, medium, and large effects, respectively.

## 3. Results

Table 1 presents the characteristics of selected somatic and performance parameters of the study group. The average age of the operators was less than 35 years, with a minimum age of 28 years and a maximum age of 42 years. The average height of the subjects was 180.4 ± 5.0 cm, while their average weight was 85.5 ± 8.1 kg, which indicates moderate homogeneity of the group in terms of somatic characteristics.

The performance parameters of the soldiers were determined based on VO_2_max, expressed in milliliters per minute (mL/min) and milliliters per kilogram of body weight per minute (mL/kg/min), as well as maximum heart rate (HRmax), expressed in beats per minute (bpm). These indicators were obtained from the 20mSRT, which allowed for the assessment of the participants’ aerobic fitness.

The average maximum oxygen uptake was 4253.1 mL, and ranged from 3497.0 to 5525.0 mL. The relative value of this parameter was close to 50 mL, with a minimum value of 41.6 mL/kg/min and a maximum value of 59.4 mL/kg/min.

Table 2 presents the results of energy expenditure indicators during the movement of soldiers at different speeds, both without a load and with an external load. As the speed increased, the values of all energy expenditure indicators clearly increased. Running at a speed of 10.5 km/h generated the highest energy cost—an average of 14.77 kcal/min without a load and 17.70 kcal/min with a backpack. This represents a more than threefold increase in energy expenditure compared to the lowest speed (4.5 km/h), for which the values were 5.01 kcal/min and 6.35 kcal/min, respectively.

A similar trend was observed in terms of the energy cost of the stage: from 25.04 kcal (without load) and 31.73 kcal (with backpack) at a speed of 4.5 km/h, to 73.87 kcal and 88.51 kcal, respectively, at the fastest speed.

When analyzing the direction of changes in individual parameters, it should be noted that the values of most indicators increased with increasing speed. The exception was fat oxidation, which increased to a speed of 8.5 km/h without a load (4.40 kcal/min) and to 6.5 km/h with a backpack (3.35 kcal/min), after which, it decreased at the highest intensity of effort. Fat burning was relatively lower when moving with a 20 kg load, which indicates a greater use of carbohydrates as the main energy substrate with increasing intensity and weight of the equipment carried.

**Figure 1:**
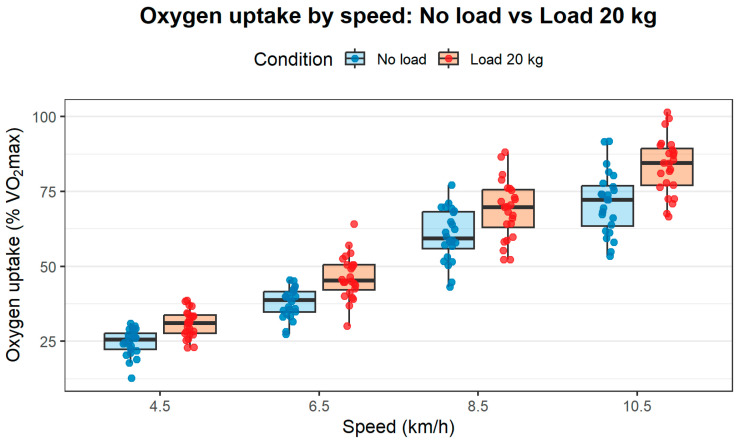
The increase in oxygen consumption across speeds, with and without a load.

Table 3 presents the results of physiological parameters obtained during the movement of soldiers at increasing speeds without a load and with an additional external load of 20 kg. With increasing speed and intensity of physical effort, a gradual increase in heart rate was observed. At a speed of 4.5 km/h, the average value was less than 48% of the maximum heart rate, while when moving with a load of 20 kg, it was more than 10% higher. At the same time, average oxygen uptake rose from 25% to 31%, representing an increase of over 20%. These values increased systematically with the increase in the speed of the operators. At the highest speed—10.5 km/h—the average value was 78.45% of the maximum heart rate without a load and 88.96% with a load of 20 kg. Oxygen uptake also increased from 71.25% to 83.51% of VO_2_max (Figure 1).

The increase in exercise intensity also resulted in an increase in oxygen consumption and carbon dioxide exhalation. When running at a speed of 10.5 km/h, the amount of carbon dioxide exhaled was more than three times higher than at the lowest speed, during both tests with and without a load. Similar relationships were observed for oxygen uptake. The increasing contribution of anaerobic processes is illustrated by the respiratory exchange ratio (RER), which, at a speed of 10.5 km/h, was 0.94 for trials performed without an additional load and 1.03 for trials with a military backpack.

Two-way repeated-measures ANOVA (Table 4) revealed a significant main effect of Speed on all dependent variables (all *p* < 0.001), with very large effect sizes (η^2^p = 0.41–0.98), indicating substantial increases in physiological and metabolic responses across speed levels. A significant main effect of external load was also observed for all variables (all *p* ≤ 0.001; η^2^p = 0.20–0.66), demonstrating higher metabolic and cardiovascular demands when wearing an external load. Significant Speed × External load interactions were found for CHO, EE, Fat, kcal/stage, VCO_2_, VO_2_ (mL/kg/min), VO_2_ (mL/min), and %VO_2_max (η^2^p = 0.08–0.35), suggesting that the effect of speed differed depending on the backpack condition for these outcomes. In contrast, no significant interactions were observed for HRmax (%) or HR (bpm) (*p* = 0.75), indicating similar heart rate responses across speeds irrespective of the external load.

## 4. Discussion

Proper nutrition is essential for soldiers to function properly and, consequently, plays an important role in achieving objectives when performing tasks in tactical operations [42]. Nutritional standards, including those for special forces, constitute a clear and concise set of key principles designed to facilitate the selection of a balanced diet, which is of great importance not only for health but also for general well-being [7]. The high level of physical fitness of special forces soldiers reflects their high level of physical activity and proper nutrition [43]. Among Polish special forces, neither underweight nor obesity is observed [43]. The results of our research confirm this. According to the BMI classification, overweight is muscular overweight because the body fat content is within the normal range. The conclusions of Polish studies confirm the validity of conducting training courses that systematize basic nutritional knowledge and training courses that take into account the specific nature and requirements of special forces soldiers [44]. Studies on the energy expenditure of soldiers suggest that the initial training of special forces is deliberately rigorous because it can be used as a model for assessing the physiological load of military operations in this unique population in a controlled environment [24]. By quantitatively defining and grouping training exercises according to physical activity factors, the energy requirements of special forces soldiers can be predicted with reasonable accuracy, which should help to avoid undesirable energy deficits and weight loss [45]. The findings of a systematic literature review indicate that military recruits worldwide are likely to consume too little energy during the extended period of initial training, with a greater deficit in carbohydrates compared to other macronutrients [46]. Research conducted by Special Operations Forces during field training, both in cold and hot conditions, also showed insufficient carbohydrate intake [47]. The results of our study indicate a greater use of carbohydrates as the main energy substrate with increasing intensity and weight of equipment carried, starting at a speed of 8.5 km/h (jogging). The observed shift in energy substrate utilization towards carbohydrate dominance with increasing exercise intensity and weight of equipment carried indicates an increased risk of rapid depletion of muscle glycogen stores. It is necessary to adequately rebuild muscle and liver glycogen stores on a daily basis in order to maintain the ability to exercise continuously and engage in intense activity [48]. From a practical point of view, this justifies the need to individualize nutritional strategies to ensure an adequate supply of carbohydrates before and during activities requiring rapid movement with a load. It is worth noting that exposure to extreme environmental conditions can further exacerbate energy deficits [49]. Research by Johnson et al. [47] showed that the total energy expenditure of Special Operations Forces soldiers was similar in hot and cold environments, but differences in energy intake were found. Energy balance varies during deployment depending on factors such as assigned duties, length of deployment or task, environment and access to food [50,51]. Although the study was conducted under controlled conditions (an athletics hall), the values obtained can serve as a reference point for estimating energy costs in field conditions. After taking into account factors such as terrain, weather conditions, and altitude above sea level, these results can be used in energy balance models for actual military operations. It is emphasized that scientific justification is still needed when developing a reference diet for military operations [52]. The energy expenditure generated provides the basis for determining the nutritional model and the amount of energy required for daily food rations [53].

The analysis was conducted at four speeds, two of which corresponded to walking (4.5 km/h and 6.5 km/h) and two to running (8.5 km/h and 10.5 km/h). The study took into account both physiological parameters and energy indicators. Statistical analysis showed that both an increase in movement speed and the use of an external load significantly increased energy expenditure and led to an increase in the analyzed physiological parameters (*p* < 0.001).

This phenomenon is consistent with earlier reports indicating that the weight of equipment carried and walking speed are key determinants of metabolic cost and soldier performance [27,31,54,55,56]. The increase in energy demand resulting from the additional load results in increased oxygen consumption, higher heart rate, and increased minute ventilation [26,31,54,56,57]. Similar relationships were observed in our own studies, in which walking with a backpack was associated with an average increase in VO_2_ of 4.45 mL/kg/min compared to exercise without external load. Importantly, at a speed of 4.5 km/h, this difference was 3.01 mL/kg/min, while at 10.5 km/h, it increased to 6.23 mL/kg/min, indicating an increasing metabolic cost with increasing speed. Similar observations were reported by Boffey et al. [56], indicating that after exceeding an intensity corresponding to approximately 47% VO_2_max, oxygen uptake and ventilation begin to increase in a non-linear manner.

Studies involving soldiers have also shown that at very high loads exceeding 40% of their body weight, parameters such as heart rate and VO_2_ increase faster than proportionally to the increase in equipment weight, and walking speed can drop sharply [31,58]. In studies by Looney et al. [31] involving US Army soldiers, at loads of 0%, 22%, 44%, and 66% of body weight, a significant increase in oxygen consumption was observed with increasing weight, from approximately 25% VO_2_max without a load to almost 50% VO_2_max with the highest load. At the same time, the heart rate increased by an average of 40–50 beats/min in the highest variant compared to walking without load [31].

During increased exercise with an additional load, especially in repeated trials, a non-linear increase in physiological parameters was also noted by Vine et al. [57] and James et al. [59]. However, it should be emphasized that in the studies by Vine et al. [57], despite a similar load (25 kg), the duration of a single trial was over 60 min. The results of James et al. [59] show that the effects of prolonged walking with a load of 32 kg can persist at least until the next day and include not only an increase in VO_2_ and VCO_2_, but also a weakening of the basic locomotor muscles. It is worth noting, however, that in the study by James et al. [59], the study group did not consist of active soldiers, but only civilians, which may have had a significant impact on the results obtained.

The vast majority of the studies cited concern the assessment of performance parameters during walking [26,31,57,58,59]. Our own studies have shown that an increase in speed is the primary factor determining the increase in the physiological parameters analyzed. At a speed of 10.5 km/h, VO_2_ uptake increased almost threefold compared to walking at 4.5 km/h. Moreover, for tests performed without an external load, these values were approximately 78% of VO_2_max, while in tests with a load of 20 kg, they exceeded 88% of VO_2_max. A similar protocol in running conditions close to 8 km/h was used by Kessels et al. [60], who observed an increase in heart rate from approximately 140 bpm to 160 bpm during a 5-min run with a load of 17.1 kg. For comparison, the average heart rate in our own studies at a speed of 8.5 km/h was slightly lower at less than 150 bpm. However, this difference may have been due to the different characteristics of the study groups, including the age of the participants and the fact that in the study by Kessels et al. [60], the subjects had no experience in carrying additional military loads.

The results suggest that special forces soldiers should be prepared to work in conditions of high energy expenditure and emphasize the importance of specialized endurance and strength training, which increases energy efficiency and load tolerance. The data obtained provides a practical basis for estimating the energy costs of combat and training tasks and can be used to optimize nutritional strategies, particularly in terms of energy and carbohydrate supply for special forces soldiers. These results are important for planning food rations, reducing energy deficits, and supporting exercise capacity under conditions of high physical exertion.

### Strengths and Limitations

Undoubtedly, the strength of the study lies in the participation of an elite group of Polish special forces soldiers. There are a limited number of published studies on energy expenditure related to the load carried and the speed of movement of soldiers. This study provides data on energy expenditure related to the speed of movement of a soldier with an additional load. However, the limitation is that the study was conducted under controlled conditions in an athletics hall, without full tactical uniforms (helmet, vest, weapon) and not in a field setting with naturally changing terrain and atmospheric conditions, which could lead to an underestimation of actual energy requirements in operational conditions. Another limitation is that the study was conducted only with male operators, which was dictated by the absence of women in the special units studied. Furthermore, the results of the study refer to an elite population of soldiers, which clearly differs from classic special units and even more so from the civilian population. Even within the elite study group, differences in physical condition could have affected individual EE values.

## 5. Conclusions

Adding a 20 kg tactical backpack causes a significant increase in energy expenditure at all speed levels. The additional load resulted in an average increase of 10% in heart rate (%HRmax) and 20% in oxygen uptake (%VO_2_max).

A more than threefold increase in energy expenditure was recorded (14.77 kcal/min without a load and 17.70 kcal/min with a backpack at a speed of 10.5 km/h vs. 5.01 kcal/min and 6.35 kcal/min at a speed of 4.5 km/h).

At low speeds, fat is the dominant source of energy, but as speed and load increase, the proportion of CHO increases, which increases the risk of rapid glycogen depletion. The results obtained can be used by those preparing nutritional guidelines and strategies for special forces soldiers based on energy expenditure.

## Figures and Tables

**Table 1:** Characteristics of selected somatic and performance indicators.

Parameter	M	Min	Max	Sd
Age (years)	34.8	28.0	42.0	4.0
BH (cm)	180.4	171.0	192.0	5.0
BW (kg)	85.5	71.2	99.0	8.1
BMI (kg/m^2^)	26.3	23.1	29.6	1.5
BF (%)	11.0	6.8	16.8	2.3
BF (kg)	9.5	5.4	16.4	2.7
FFM (kg)	76.0	64.9	85.6	6.3
TBW (kg)	51.9	44.1	59.4	4.3
Distance (m)	2059.2	1520.0	2420.0	245.9
HRmax (bpm)	186.4	174.0	198.0	6.3
VO_2_max (mL/min)	4253.1	3497.0	5525.0	510.8
VO_2_max (mL/kg/min)	49.9	41.6	59.4	5.5
RER	1.22	1.12	1.30	0.06

M—Mean; Sd—standard deviation; Min—Minimum; Max—Maximum; BH—body height; BW—body weight; BMI—body mass index; BF—body fat; FFM—fat-free mass; TBW—total body water; VO_2_max—maximal oxygen uptake; HRmax—maximal heart rate; RER—Respiratory Exchange Ratio.

**Table 2:** Energy expenditure indicators during movement of soldiers at different speeds without a load and with an external load of 20 kg.

Parameter	Speed (km/h)	No Equipment Load		Equipment Load (20 kg)	
		M	Sd	M	Sd
CHO (kcal/min)	4.5	1.88	1.18	4.33	1.01
	6.5	3.77	1.24	6.17	1.56
	8.5	7.97	1.98	11.88	2.27
	10.5	11.84	2.58	16.65	2.20
EE (kcal/min)	4.5	5.01	0.74	6.35	0.48
	6.5	7.68	0.69	9.52	0.77
	8.5	12.37	1.09	14.36	1.38
	10.5	14.77	1.46	17.70	1.32
Fat (kcal/min)	4.5	3.13	1.13	2.01	1.04
	6.5	3.91	1.09	3.35	1.65
	8.5	4.40	1.74	2.48	1.96
	10.5	2.93	2.03	1.05	1.79
kcal/stage	4.5	25.04	3.72	31.73	2.41
	6.5	38.41	3.47	47.62	3.87
	8.5	61.86	5.43	71.81	6.91
	10.5	73.87	7.28	88.51	6.62

M—Mean; Sd—standard deviation; CHO (kcal/min)—carbohydrate oxidation; EE (kcal/min)—Energy expenditure; Fat (kcal/min)—fat oxidation; kcal/stage—Energy expenditure per stage (5 min).

**Table 3:** Physiological indicators during movement of soldiers at different speeds without a load and with an external load of 20 kg.

Parameter/Stage	Speed (km/h)	No Equipment Load		Equipment Load (20 kg)	
		M	Sd	M	Sd
HRmax (%)	4.5	47.78	4.83	58.54	5.08
	6.5	56.49	5.10	66.35	5.90
	8.5	69.69	5.98	80.25	6.04
	10.5	78.45	6.26	88.96	5.65
HR (bpm)	4.5	89.00	8.62	109.08	9.44
	6.5	105.25	9.27	123.63	10.86
	8.5	129.83	10.81	149.54	11.45
	10.5	146.13	11.16	165.75	10.59
RER	4.5	0.81	0.07	0.91	0.05
	6.5	0.85	0.04	0.90	0.05
	8.5	0.90	0.04	0.96	0.05
	10.5	0.94	0.05	1.03	0.08
VCO_2_ (mL/min)	4.5	851.50	146.09	1177.29	93.40
	6.5	1350.83	144.34	1747.21	148.13
	8.5	2267.58	227.30	2774.75	288.81
	10.5	2824.08	316.67	3627.83	318.55
VO_2_ (mL/min)	4.5	1043.79	153.47	1295.04	102.72
	6.5	1588.63	140.53	1949.13	163.87
	8.5	2532.88	220.26	2899.79	282.13
	10.5	2992.00	290.84	3515.88	276.02
VO_2_ (mL/kg/min)	4.5	12.21	1.56	15.22	1.34
	6.5	18.64	1.39	22.93	2.28
	8.5	29.71	2.13	33.99	2.57
	10.5	35.06	2.28	41.29	3.00
%VO_2_max (mL/kg/min)	4.5	24.81	4.39	30.88	4.52
	6.5	37.79	4.95	46.53	7.25
	8.5	60.33	8.61	68.98	9.78
	10.5	71.25	10.44	83.51	9.53

M—Mean; Sd—standard deviation; HRmax (%)—percentage of maximal heart rate; HR (bpm)—heart rate; RER—Respiratory Exchange Ratio; VCO_2_ (mL/min)—Volume of carbon dioxide output; VO_2_ (mL/min)—Volume of oxygen uptake; VO_2_ (mL/kg/min)—Oxygen uptake per kilogram of body mass, %VO_2_max (mL/kg/min)—percentage of maximal oxygen uptake.

**Table 4:** Two-way ANOVA results for energy expenditure indicators and physiological indicators.

Variable	Effect	F (df)	*p*	η^2^p
CHO (kcal/min)	Speed	903.6 (3)	<0.001	0.952
	External load	58.5 (1)	<0.001	0.560
	Speed × External load	12.3 (3)	<0.001	0.212
EE (kcal/min)	Speed	2316.8 (3)	<0.001	0.981
	External load	65.0 (1)	<0.001	0.586
	Speed × External load	11.4 (3)	<0.001	0.199
Fat (kcal/min)	Speed	32.62 (3)	<0.001	0.415
	External load	11.8 (1)	0.001	0.203
	Speed × External load	5.96 (3)	<0.001	0.115
kcal/stage	Speed	2251.32 (3)	<0.001	0.980
	External load	55.7 (1)	<0.001	0.548
	Speed × External load	4.14 (3)	0.008	0.082
HRmax (%)	Speed	1959.96 (3)	<0.001	0.977
	External load	46.1 (1)	<0.001	0.501
	Speed × External load	0.40 (3)	0.750	0.009
HR (bpm)	Speed	1963.87 (3)	<0.001	0.977
	External load	48.0 (1)	<0.001	0.510
	Speed × External load	0.42 (3)	0.740	0.009
VCO_2_ (mL/min)	Speed	2160.1 (3)	<0.001	0.979
	External load	88.7 (1)	<0.001	0.658
	Speed × External load	24.6 (3)	<0.001	0.348
VO_2_ (mL/kg/min)	Speed	2436.42 (3)	<0.001	0.981
	External load	84.4 (1)	<0.001	0.647
	Speed × External load	8.91 (3)	<0.001	0.162
VO_2_ (mL/min)	Speed	2145.04 (3)	<0.001	0.979
	External load	53.7 (1)	<0.001	0.539
	Speed × External load	7.72 (3)	<0.001	0.144
%VO_2_max (mL/kg/min)	Speed	23,687.4 (3)	<0.001	0.972
	External load	19.3 (1)	<0.001	0.295
	Speed × External load	77.4 (3)	0.002	0.101

F (df)—F statistic with degrees of freedom; *p*—significance level; η^2^p—partial eta squared; HRmax (%)—percentage of maximal heart rate; CHO (kcal/min)—carbohydrate oxidation; EE (kcal/min)—Energy expenditure; Fat (kcal/min)—fat oxidation; kcal/stage—Energy expenditure per stage (5 min); HR (bpm)—heart rate; VCO_2_ (mL/min)—Volume of carbon dioxide output; VO_2_ (mL/min)—Volume of oxygen uptake; VO_2_ (mL/kg/min)—Oxygen uptake per kilogram of body mass, %VO_2_max (mL/kg/min)—percentage of maximal oxygen uptake.

## Data Availability

The data presented in this study are available on request from the corresponding author due to ethical restrictions related to participant confidentiality. All data generated or analyzed during this study are included in the article.

## Abbreviations

The following abbreviations are used in this manuscript:

BH	Body height
BW	Body weight
VO_2_max	Maximal oxygen uptake
%VO_2_max	Percentage of maximal oxygen uptake
HRmax	Maximal heart rate
20mSRT	20-metre Shuttle Run Test
VO_2_	Volume of oxygen uptake
VCO_2_	Volume of carbon dioxide output
EE	Energy expenditure
RER	Respiratory Exchange Ratio
mL/min	Milliliters per minute
mL/kg/min	Milliliters of oxygen per kilogram of body mass per minute
Bpm	Beats per minute

## References

[1] Garg S. (2025). The Importance of Physical Fitness and Health for Soldiers: The Role of Military Doctors. Univers. Res. Rep..

[2] Flood A., Keegan R.J. (2022). Cognitive Resilience to Psychological Stress in Military Personnel. Front. Psychol..

[3] Sporiš G., Harasin D., Bok D., Matika D., Vuleta D. (2012). Effects of a Training Program for Special Operations Battalion on Soldiers’ Fitness Characteristics. J. Strength Cond. Res..

[4] Schulze C., Lindner T., Goethel P., Müller M., Kundt G., Stoll R., Mittelmeier W., Bader R. (2015). Evaluation of the Physical Activity of German Soldiers Depending on Rank, Term of Enlistment, and Task Area. Mil. Med..

[5] Lisowski V.O., Mihuta I.Y. (2013). Importance of Coordination Skills Essential Psychophysical Demonstrated Competencies as a Military Specialists. Phys. Educ. Stud..

[6] Martins L.C.X., Lopes C.S. (2013). Rank, Job Stress, Psychological Distress and Physical Activity among Military Personnel. BMC Public Health.

[7] Bertrandt J., Anyżewska A., Tomczak A., Szarska E., Maculewicz E., Marszałkowska J., Lepionka T., Gaździńska A., Kłos A., Wojskowy Instytut Higieny i Epidemiologii im. gen. Karola Kaczkowskiego (Warszawa) (2020). Normy Żywienia dla Żołnierzy Sił Zbrojnych RP oraz Funkcjonariuszy Służb Mundurowych (Wojsk Lądowych, Marynarki Wojennej, Sił Powietrznych, Wojsk Specjalnych, Straży Granicznej i Policji) Uwzględniające Specyfikę i Charakter Służby, Stanowiące Podstawę Planowania i Realizacji Żywienia w Warunkach Garnizonowych i Poligonowych.

[8] Gijsbertse K., Linssen L., Woering A., Catoire M. (2021). The Effects of Mass, Bulk and Stiffness of Personal Protective Equipment and Clothing on Physical Performance When Performing a Military Mobility Obstacle Course. Appl. Ergon..

[9] Bossi L.L.M., Jones M.L.H., Kelly A., Tack D.W. (2016). A Preliminary Investigation of the Effect of Protective Clothing Weight, Bulk and Stiffness on Combat Mobility Course Performance. Proc. Hum. Factors Ergon. Soc. Annu. Meet..

[10] Brown S., Mitchell K.B. (2019). Preliminary Development of an Integrated Mobility, Lethality, and Survivability Soldier Performance Testing Platform. Advances in Human Factors and Systems Interaction, Proceedings of the AHFE.

[11] Orr R.M., Pope R., Coyle J., Johnston V. (2015). Occupational Loads Carried by Australian Soldiers on Military Operations. J. Health Saf. Environ..

[12] Birrell S.A., Hooper R.H., Haslam R.A. (2007). The Effect of Military Load Carriage on Ground Reaction Forces. Gait Posture.

[13] Knapik J.J., Harman E.A., Steelman R.A., Graham B.S. (2012). A Systematic Review of the Effects of Physical Training on Load Carriage Performance. J. Strength Cond. Res..

[14] Sell T.C., Chu Y., Abt J.P., Nagai T., Deluzio J., McGrail M.A., Rowe R.S., Lephart S.M. (2010). Minimal Additional Weight of Combat Equipment Alters Air Assault Soldiers’ Landing Biomechanics. Mil. Med..

[15] Roy T.C., Knapik J.J., Ritland B.M., Murphy N., Sharp M.A. (2012). Risk Factors for Musculoskeletal Injuries for Soldiers Deployed to Afghanistan. Aviat. Space Environ. Med..

[16] Knapik J.J., Reynolds K.L., Harman E. (2004). Soldier Load Carriage: Historical, Physiological, Biomechanical, and Medical Aspects. Mil. Med..

[17] Li S.S.W., Chan O.H.T., Ng T.Y., Kam L.H., Ng C.Y., Chung W.C., Chow D.H.K. (2019). Effects of Backpack and Double Pack Loads on Postural Stability. Ergonomics.

[18] Liu B.-S. (2007). Backpack Load Positioning and Walking Surface Slope Effects on Physiological Responses in Infantry Soldiers. Int. J. Ind. Ergon..

[19] Sessoms P.H., Gobrecht M., Niederberger B.A., Sturdy J.T., Collins J.D., Dominguez J.A., Jaworski R.L., Kelly K.R. (2020). Effect of a Load Distribution System on Mobility and Performance during Simulated and Field Hiking While under Load. Ergonomics.

[20] Genitrini M., Dotti F., Bianca E., Ferri A. (2022). Impact of Backpacks on Ergonomics: Biomechanical and Physiological Effects: A Narrative Review. Int. J. Environ. Res. Public Health.

[21] Huang T.-W.P., Kuo A.D. (2014). Mechanics and Energetics of Load Carriage during Human Walking. J. Exp. Biol..

[22] Pal M., Yadav A., Arya K., Chatterjee T., Bhattacharyya D., Kumar B. (2020). Optimization of Load Carriage at Desert Environment. Int. J. Ind. Ergon..

[23] Scott P.A., Christie C.J. (2004). “Optimal” Speed–Load Combinations for Military Manoeuvres. Int. J. Ind. Ergon..

[24] Margolis L.M., Crombie A.P., McClung H.L., McGraw S.M., Rood J.C., Montain S.J., Young A.J. (2014). Energy Requirements of US Army Special Operation Forces during Military Training. Nutrients.

[25] Tharion W.J., Lieberman H.R., Montain S.J., Young A.J., Baker-Fulco C.J., Delany J.P., Hoyt R.W. (2005). Energy Requirements of Military Personnel. Appetite.

[26] Chatterjee S., Chatterjee T., Bhattacharyya D., Sen S., Pal M. (2018). Effect of Heavy Load Carriage on Cardiorespiratory Responses with Varying Gradients and Modes of Carriage. Mil. Med. Res..

[27] Grenier J.G., Peyrot N., Castells J., Oullion R., Messonnier L., Morin J.-B. (2012). Energy Cost and Mechanical Work of Walking during Load Carriage in Soldiers. Med. Sci. Sports Exerc..

[28] Lindner T., Schulze C., Woitge S., Finze S., Mittelmeier W., Bader R. (2012). The Effect of the Weight of Equipment on Muscle Activity of the Lower Extremity in Soldiers. Sci. World J..

[29] Pal M.S., Majumdar D., Pramanik A., Chowdhury B., Majumdar D. (2014). Optimum Load for Carriage by Indian Soldiers on Different Uphill Gradients at Specified Walking Speed. Int. J. Ind. Ergon..

[30] Elias S. (2021). Predictive Thin Plate Spline Model for Estimation of Load Carriage at Varying Gradient and Speed. Def. Life Sci. J..

[31] Looney D.P., Doughty E.M., Figueiredo P.S., Vangala S.V., Pryor J.L., Santee W.R., McClung H.L., Potter A.W. (2021). Effects of Modern Military Backpack Loads on Walking Speed and Cardiometabolic Responses of US Army Soldiers. Appl. Ergon..

[32] Graham S.M., Florida-James G.D., Connaboy C., Clement R., Simpson R.J. (2009). Metabolic Differences Between Backpack Walking and Running at A Fixed Treadmill Velocity in ‘Elite’ British Soldiers: A Pilot Study: 708: May 28 8:15 AM–8:30 AM. Med. Sci. Sports Exerc..

[33] Faghy M.A., Shei R.-J., Armstrong N.C.D., White M., Lomax M. (2022). Physiological Impact of Load Carriage Exercise: Current Understanding and Future Research Directions. Physiol. Rep..

[34] Paśko W., Guła P., Brożyna M., Dziadek B., Zadarko E., Śliż M., Polak K., Przednowek K. (2022). Psychomotor Abilities of Candidates for Polish Special Forces. Sci. Rep..

[35] Brown T.N., O’Donovan M., Hasselquist L., Corner B.D., Schiffman J.M. (2014). Body Borne Loads Impact Walk-to-Run and Running Biomechanics. Gait Posture.

[36] Coombes J.S., Kingswell C. (2005). Biomechanical and Physiological Comparison of Conventional Webbing and the M83 Assault Vest. Appl. Ergon..

[37] Walsh G.S., Low D.C. (2021). Military Load Carriage Effects on the Gait of Military Personnel: A Systematic Review. Appl. Ergon..

[38] Léger L.A., Mercier D., Gadoury C., Lambert J. (1988). The Multistage 20 Metre Shuttle Run Test for Aerobic Fitness. J. Sports Sci..

[39] Weir J.B.D.B. (1949). New Methods for Calculating Metabolic Rate with Special Reference to Protein Metabolism. J. Physiol..

[40] Frayn K.N. (1983). Calculation of Substrate Oxidation Rates in Vivo from Gaseous Exchange. J. Appl. Physiol..

[41] Péronnet F., Massicotte D. (1991). Table of Nonprotein Respiratory Quotient: An Update. Can. J. Sport Sci. J. Can. Sci. Sport.

[42] Kler P. (2019). Standardy żywienia wojsk w działaniach taktycznych podstawą właściwej realizacji zadań. Gospod. Mater. Logistyka.

[43] Tomczak A., Bertrandt J., Kłos A., Bertrandt B. (2014). Assessment of Physical Fitness, Physical Capacity and Nutritional Status of Soldiers Serving in the “GROM” Polish Special Forces Unit. Probl. Hig. Epidemiol.

[44] Bębnowicz A., Bertrandt B., Kler P., Bertrandt J. (2015). Ocena poziomu wiedzy żywieniowej żołnierzy pełniących służbę w polskiej jednostce Wojsk Specjalnych “GROM”. Probl. Hig. Epidemiol.

[45] Barringer N.D., Pasiakos S.M., McClung H.L., Crombie A.P., Margolis L.M. (2018). Prediction Equation for Estimating Total Daily Energy Requirements of Special Operations Personnel. J. Int. Soc. Sports Nutr..

[46] Baker B.A., Cooke M.B., Belski R., Carins J.E. (2020). The Influence of Training on New Army Recruits’ Energy and Macronutrient Intakes and Performance: A Systematic Literature Review. J. Acad. Nutr. Diet..

[47] Johnson C.D., Simonson A.J., Darnell M.E., DeLany J.P., Wohleber M.F., Connaboy C. (2018). Energy Expenditure and Intake during Special Operations Forces Field Training in a Jungle and Glacial Environment. Appl. Physiol. Nutr. Metab. Physiol. Appl. Nutr. Metab..

[48] Murray B., Rosenbloom C. (2018). Fundamentals of Glycogen Metabolism for Coaches and Athletes. Nutr. Rev..

[49] Margolis L.M., Pasiakos S.M. (2023). Performance Nutrition for Cold-Weather Military Operations. Int. J. Circumpolar Health.

[50] O’Leary T.J., Wardle S.L., Greeves J.P. (2020). Energy Deficiency in Soldiers: The Risk of the Athlete Triad and Relative Energy Deficiency in Sport Syndromes in the Military. Front. Nutr..

[51] Tryon E.G., Barringer N.D., Lieberman H.R., Conkright W.R., Tryon E.G., Barringer N.D., Lieberman H.R., Conkright W.R. (2024). Energy Deficit and Factors Associated with Energy Balance during a Combat Deployment in U.S. Army Special Operation Forces Soldiers. Nutrients.

[52] Mizushima R., Miyachi M., Yoshimura E., Hatamoto Y., Matsumoto M., Hamada Y., Hatanaka M., Maeno A., Shimomura C., Takimoto H. (2024). Dietary Reference Intake for Military Operations: A Scoping Review. PeerJ.

[53] Anyżewska A., Łakomy R., Lepionka T., Szarska E., Maculewicz E., Tomczak A., Bertrandt J. (2020). Association Between Diet, Physical Activity and Body Mass Index, Fat Mass Index and Bone Mineral Density of Soldiers of the Polish Air Cavalry Units. Nutrients.

[54] Arcidiacono D.M., Lavoie E.M., Potter A.W., Vangala S.V., Holden L.D., Soucy H.Y., Karis A.J., Friedl K.E., Santee W.R., Looney D.P. (2023). Peak Performance and Cardiometabolic Responses of Modern US Army Soldiers during Heavy, Fatiguing Vest-Borne Load Carriage. Appl. Ergon..

[55] Looney D.P., Lavoie E.M., Notley S.R., Holden L.D., Arcidiacono D.M., Potter A.W., Silder A., Pasiakos S.M., Arellano C.J., Karis A.J. (2024). Metabolic Costs of Walking with Weighted Vests. Med. Sci. Sports Exerc..

[56] Boffey D., Harat I., Gepner Y., Frosti C.L., Funk S., Hoffman J.R. (2019). The Physiology and Biomechanics of Load Carriage Performance. Mil. Med..

[57] Vine C.A.J., Coakley S.L., Blacker S.D., Runswick O.R., Myers S.D. (2024). Metabolic, Cardiovascular, Neuromuscular and Perceptual Responses to Repeated Military-Specific Load Carriage Treadmill Simulations. Eur. J. Sport Sci..

[58] Looney D.P., Doughty E.M., Marrero A.S., Figueiredo P.S., Welles A.P., Santee W.R., McClung H.L., Sanford D.P., Potter A.W. (2020). Cardiovascular And Metabolic Responses Of US Army Soldiers During Heavy Military Rucksack Carriage: 3904 Board #221 May 30 9:00 AM–10:30 AM. Med. Sci. Sports Exerc..

[59] James S., Damian C., Mathew B. (2021). Energy Cost and Knee Extensor Strength Changes Following Multiple Day Military Load Carriage. Appl. Ergon..

[60] Kessels I., Koopman B., Verdonschot N., Marra M., Gijsbertse K. (2021). The Added Value of Musculoskeletal Simulation for the Study of Physical Performance in Military Tasks. Sensors.

